# The Antipsychotic Medication Management Fidelity Scale: Psychometric properties

**DOI:** 10.1007/s10488-020-01018-1

**Published:** 2020-02-06

**Authors:** Torleif Ruud, Karin Drivenes, Robert E. Drake, Vegard Øksendal Haaland, Matthew Landers, Bjørn Stensrud, Kristin S. Heiervang, Lars Tanum, Gary R. Bond

**Affiliations:** 1 Division of Mental Health Services, Akershus University Hospital, Lørenskog, Norway; 2 Institute of Clincial Medicine, University of Oslo, Oslo, Norway; 3 Division of Mental Health, Sørlandet Hospital, Kristiansand, Norway; 4 South Eastern Norway Hospital Pharmacy Enterprise, Kristiansand, Norway; 5 Westat, Lebanon, NH USA; 6 Sørlandet Hospital, Kristiansand, Norway; 7 Clinical Neuroscience Research Group, Department of Psychology, The Faculty of Social Sciences, University of Oslo, Oslo, Norway; 8 Duke University, Durham, NC USA; 9 Division of Mental Health, Innlandet Hospital Trust, Brumunddal, Norway; 10 National Advisory Unit on Concurrent Substance Abuse and Mental Health Disorders, Brumunddal, Norway; 11 Oslo Metropolitan University, Oslo, Norway

**Keywords:** Psychosis, Antipsychotic medication, Implementation, Evidence-based practice, Fidelity scale

## Abstract

The paper describes the Antipsychotic Medication Management Fidelity Scale and its psychometric properties, including interrater reliability, frequency distribution, sensitivity to change and feasibility. Fidelity assessors conducted fidelity reviews four times over 18 months at eight sites receiving implementation support for evidence-based antipsychotic medication management. Data analyses shows good to fair interrater reliability, adequate sensitivity to change over time and good feasibility. At 18 months, item ratings varied from poor to full fidelity on most items. Use of the scale can assess fidelity to evidence-based guidelines for antipsychotic medication management and guide efforts to improve practice. Further research should improve and better calibrate some items, and improve the procedures for access to information.

Trial registration: ClinicalTrials.gov Identifier: NCT03271242.

**Electronic supplementary material:**

The online version of this article (10.1007/s10488-020-01018-1) contains supplementary material, which is available to authorized users.

## Introduction

Clinical trials and systematic reviews have established the core components of evidence-based antipsychotic medication management. Clinical guidelines make this research-based knowledge more available for clinicians. However, implementation of clinical guidelines remains fraught with difficulties (Barbui et al. 2014), often because guidelines lack sufficient details and measures.

Antipsychotic medication management requires an operational model such as a fidelity scale. Fidelity scales measure evidence-based practices and provide objective data to a clinical team or program regarding implementation of key components of the model (Bond and Drake 2019). Fidelity scales also facilitate research to identify, measure, and improve components and effects. We therefore developed and tested a fidelity scale for antipsychotic medication management for use in treatment of persons with psychoses within the diagnostic groups F20–F29 in ICD-10, with schizophrenia as the major group often needing long term antipsychotic medication.

### Earlier Fidelity Scales for Antipsychotic Medication Management

We found one previously published fidelity scale for antipsychotic medication management, the MedMAP Fidelity Scale (Taylor et al. 2009). It contained 1 prescriber fidelity scale (23 items) based on assessment of documentation in patient records and 1 organizational fidelity scale (17 items) based on assessment of established procedures instructing clinical practice. The domains and items were developed by consensus of 11 experts. The scales showed good psychometric properties (e.g. good interrater reliability for the total scale and most items), which were replicated in another study (Howard et al. 2009). However, it proved to be too time-consuming and impractical for use in routine clinical practice (El-Mallakh et al. 2014), and it has not become a widely used or a well established fidelity scale. The MedMAP fidelity scales covered the core components for antipsychotic medication described below. But it also contained many items on illness and medication history and on details only relevant for some patients, which required assessment of more patient records. We concluded that there was a need for an antipsychotic medication management fidelity scale requiring less demanding work to complete and where all items were relevant for all patients prescribed antipsychotic medication.

### Aims

The aims of this study were to develop a fidelity scale to measure antipsychotic medication management and to evaluate its psychometric properties, including interrater reliability, frequency distribution, sensitivity to change and feasibility.

## Methods

### Overview

Development of the Antipsychotic Medication Management Fidelity Scale and testing its psychometric properties were part of a study on implementation of four evidence-based practices for treatment of patients with psychoses in mental health services (ClinicalTrials NCT03271242). Eight sites from four health trusts in Norway, assigned by randomization, received support to implement evidence-based antipsychotic medication management. Prior to the study, all sites were providing antipsychotic medication management, but without support for following evidence-based guidelines. The Regional Committee for Medical and Health Research Ethics approved the study (REK 2015/2169), which followed the principles in the Declaration of Helsinki. Later papers on results of the study will analyze and report the relationships between implementation support and fidelity, and the relationship between fidelity and patient outcome and experiences.

### Core Components for Antipsychotic Medication Management

Below (and in Table 1) we briefly describe 10 evidence-based core components of antipsychotic medication management, based on reviews and studies on the effect of such components.

**Table 1:** Evidence for core components of antipsychotic medication management

Components of antipsychotic medication management	Scale items	Key references to evidence
Shared decision-making	1, 7	Beitinger et al. (2014), Chewning et al. (2012) and Slade (2017)
Somatic assessment	15	De Hert et al. (2010)
Choice of antipsychotic medication	10	Leucht et al. (2013)
Dosage of antipsychotic medication	11	Oosthuizen et al. (2001) and Rothe et al. (2018)
Limiting polypharmacy	9	Gallego et al. (2012), Goodwin et al. (2009), Iversen et al. (2018) and Zink et al. (2010)
List of current medication and doses	2, 8	Buchanan et al. (2010)
Monitoring and improving adherence	3, 14	Alhewiti, (2014) and Beck et al. (2011)
Systematic measurement of symptoms	4, 12	Gharabawi et al. (2006)
Monitoring side effects	5, 13	Crowe et al. (2011), McCann et al. (2009) and van Strien et al. (2015)
Monitoring discontinuation of medication	6	Gitlin et al. (2001) and Wunderink et al. (2007)

#### Shared Decision-Making

There is an increasing emphasis on patients’ preferences and shared decision-making regarding medication and other treatments in mental health services (Slade 2017). An increasing majority of patients prefer sharing decision roles (Chewning et al. 2012), and some studies have shown positive results on patient involvement and adherence also among patients with schizophrenia and related disorders (Beitinger et al. 2014).

#### Somatic Assessment

Before starting an antipsychotic medication, clinicians should evaluate the risk of somatic co-morbidity, individual vulnerability and premature death related to serious mental illness and medication (De Hert et al. 2010).

#### Choice of Antipsychotic Medication

Clinicians and patients should consider phase of illness, clinical course, individual characteristics and preferences, and earlier experiences with antipsychotic drugs. Choosing a medication should include individual needs, preferences, and vulnerabilities (Leucht et al. 2013).

#### Dosage of Antipsychotic Medication

Clinicians and patients should consider type and phase of illness and earlier patient experiences to determine dose. Standard guidelines indicate lower doses for first episodes of psychosis, higher doses for relapse episodes (Oosthuizen et al. 2001; Rothe et al. 2018), and dose reductions during maintenance treatments.

#### Limiting Polypharmacy

Use of more than one antipsychotic drug simultaneously does not add benefits (Zink et al. 2010), but does increase the burden of side effects (Iversen et al. 2018). Polypharmacy should be a last-resort treatment option after monotherapy with two different antipsychotics plus clozapine has been ineffective (Gallego et al. 2012; Goodwin et al. 2009).

#### List of Current Medications and Doses

An easily accessible, current list of medications should be available to facilitate effective care, treatment continuity, and communication among providers (Buchanan et al. 2010).

#### Monitoring and Improving Adherence

Because adherence to antipsychotic medication is generally poor, but manageable, clinicians should monitor and address adherence (Alhewiti 2014; Beck et al. 2011).

#### Systematic Measurement of Symptoms

Clinicians should use standardized scales to assess the efficacy of antipsychotic medications. Patients who experience reduction in psychotic symptoms are more satisfied with treatment and have better compliance (Gharabawi et al. 2006).

#### Monitoring Side Effects

At least half of patients with schizophrenia on antipsychotic medication experience distressing side effects (McCann et al. 2009). Clinicians should use standardized side effect scales (van Strien et al. 2015) and also assess and address the patient’s perceptions of side effects (Crowe et al. 2011).

#### Monitoring Discontinuation of Medication

Because relapses often occur after discontinuation an antipsychotic medication, especially during the first year (Wunderink et al. 2007) but also during the second year (Gitlin et al. 2001), clinicians should taper the medication slowly and follow patients closely for 2 years after discontinuation.

### Development of the Fidelity Scale for Antipsychotic Medication

Following standardized procedures (Bond et al. 2000), we identified core components of evidence-based antipsychotic medication management from current research reviews. Table 1 shows these components, the related items in the fidelity scale, and key references documenting evidence. For each component we defined one or two items, and for each item we defined operationalized criteria and rules for rating each item on five steps from no to full fidelity. We discussed this draft version of the fidelity scale with clinicians, leaders and researchers, and then made final adjustments based on their input and on informal pilot testing in some sites.

### Sites

The eight study sites were in four health trusts in urban and rural areas of Norway. Six of the sites were community mental health centers, one was an inpatient hospital ward for patients with psychosis and drug abuse problems, and one was a high-security inpatient ward.

### Procedures

The sites received training and support to help implementation. An average of 10 leaders and clinicians from each site participated in a workshop with Norwegian experts on antipsychotic medication management. The research team developed a toolkit and distributed it to the sites. This toolkit included a description of the evidence-based practice, key literature, presentations from the workshop, and rating scales for clinical use. Implementation trainers offered in-person implementation support biweekly for 6 months and then monthly for an additional 12 months.

Trained fidelity assessors, using fidelity guidelines, conducted assessments and provided feedback to each site at baseline, and after 6, 12, and 18 months. The assessors conducted interviews with leaders and clinicians, reviewed written documentation of procedures, and reviewed 10 randomly chosen patient records. The two assessors made independent fidelity ratings, compared ratings, resolved discrepancies through discussion to reach consensus, and recorded independent and consensus ratings.

### Measures

#### Antipsychotic Medication Management Fidelity Scale

The Antipsychotic Medication Management Fidelity Scale includes 15 items, each rated on a 5-point behaviorally anchored rating scale. Most items have 4–5 specific criteria and rules for rating based on number of criteria met. The total scale includes two subscales: Policies (6 items, Items 1–6) and Prescriber Practices (9 items, Items 7–15). Fidelity assessors rate the Policies items based on semi-structured interviews with leaders and key clinicians on policies and procedures, and on reviewing written policies or procedures. The assessors rate the Prescriber Practices items based on information in 10 randomly selected patient records, including progress notes and prescription orders over the previous three months for inpatients and the previous six months for outpatients. The items are rated according to the number of criteria met, using a summary sheet and following the calculation rules described for each item in the fidelity scale. The scoring for the scale and subscales is calculated as the unweighted sum of the item ratings divided by the total number of items. The fidelity scale with instructions is available as an Online Appendix. Table 2 contains abbreviated names of items.

**Table 2:** Percentage exact agreement and interrater reliability^a^ for items based on two raters’ rating independently 8 sites 4 times for Items 1–6 and altogether 57 patient records for Items 7–15

Item	Item (short titles)	Agreement (%)	ICC	Kappa
Policies Subscale items				
1	Shared decision-making policy	66	0.75	
2	Access to medication list	69	0.85	
3	Monitor and improve adherence	81	0.76	
4	Monitor effect of medication	72	0.93	
5	Monitor side effects of medication	56	0.87	
6	Monitor clinical course after medication	69	0.88	
Policies Subscale items average		69	0.84	
Prescriber Practices Subscale items				
7	Medication decisions and patient preferences	81		0.61
8	List of medications and dose levels are updated	89		0.67
9	Polypharmacy only during change of drug	86		0.69
10	Choice of drug according to guidelines	70		0.36
11	Dosage of drug according to guidelines	79		0.51
12	Systematic monitoring of symptoms	95		− 0.02
13	Systematic monitoring of side effects	77		0.46
14	Medication adherence support	81		0.61
15	Somatic assessment at start of medication	91		0.57
Prescriber Practices Subscale items average		83		0.50
Average for all items		77		

^a^ICC for items rated 1–5 and Cohen’s kappa for patient records rated passed/failed

#### Feasibility Survey

After the final assessments, the fidelity assessors completed an online survey on their experiences with the fidelity scale. Questions included if the scale was clearly set out and with good instructions, if it was easy to get information and if it was easy to rate. Additional questions addressed the usefulness of different sources of information and the instructions for using the scale.

### Data Analyses

We had independent fidelity ratings (1–5) by the two fidelity assessors on the Policies items (Items 1–6) across 32 assessments (8 sites each rated 4 times). For these items and the subscale we calculated the intraclass correlation coefficient (ICC; McGraw and Wong 1996) based on a one-way random effects analysis of variance model for agreement between two assessors. For the items we also calculated percentage exact agreement. For ICC defined as above, we interpreted the degree of interrater reliability as suggested by Koo and Li (2016) with the levels as poor (below 0.50), moderate (0.50 to 0.74), good (0.75 to 0.90) and excellent (above 0.90).

The fidelity assessors did not make independent ratings for the Prescriber Practices items (Items 7–15). Instead, in order to determine interrater reliability, the assessors independently rated a subset of two patient records at each fidelity site visit. Thus from the 32 fidelity assessments we obtained independent dichotomous judgments (passed/failed for each item) for 57 patient records (usually 2 at each site visit) reviewed independently by both assessors. The two assessors divided the other 8 randomly selected patient records at the site between them to save time and still obtain ratings on 10 patient records. The assessors then jointly reached a consensus fidelity rating of Items 7–15 at each site. Based on the 57 pairs of independent ratings we calculated percentage exact agreement and Cohen’s Kappa for the 9 Prescriber Practices items. To rate each Prescriber Subscale item, assessors first made dichotomous ratings for each patient record on either 4 or 5 specific criteria. We also calculated interrater agreement on these criteria (See Online Appendix, Table 4). For kappa we interpreted the degree of interrater reliability as suggested in the guidelines by Cicchetti (1994) with the levels as poor (below 0.40), fair (0.40 to 0.59), good (0.60 to 0.74) and excellent (0.75 and above).

After assessing interrater agreement and reliability, we used consensus ratings in all subsequent analyses. To estimate internal consistency of the two subscales and the total scale, we used Cronbach’s alpha, calculating an alpha coefficient for each assessment. For alpha we interpreted the degree of internal consistency as suggested in the guidelines by Cicchetti (1994) with the levels as unacceptable (below 0.70), fair (0.70 to 0.79), good (0.80 to 0.89) and excellent (0.90 and above).

We next examined the item distributions at 18 months, examining mean, standard deviation, and distribution of scores across sites for full (rating = 5), adequate (4), and poor (1–3) fidelity. We also examined the distribution of site scores at 18 months.

Next, we examined the longitudinal pattern of fidelity graphically and statistically for the total scale and the two subscales. We examined the pattern in change over time using one-way ANOVA repeated measures with pairwise post hoc tests with Bonferroni corrections between baseline and 6 months, and between 6 and 18 months. We also analyzed sensitivity of change in fidelity from baseline to 18 months using paired t-tests for each item, the total scale and the two subscales, including reporting means and standard deviations at baseline and 18 months. Change over time was estimated by calculating the standardized mean difference effect size (Cohen’s d_z_) for within-subjects design (Lakens 2013). We interpreted the sensitivity to change as adequate if the improvement was statistically significant and with at least a moderate effect size (Cohen’s d_z_ ≥ 0.50).

Finally, we calculated the Pearson correlation coefficient between the Policies Subscale and the Prescriber Practices Subscale across the sites for each of the four times of assessment. We interpreted the correlation coefficients according to guidelines suggested by Schober et al. (2018).

From the feasibility survey we reported how much time the fidelity assessors on average spent on a fidelity visit, and their experiences with using the fidelity scale. We are not aware of any established measure for feasibility, but we interpreted feasibility to be good for a scale quality (clearly set out, easy to get information, easy to rate, good instruction, good instructions for preparations) if more than 60% of the fidelity assessors rated agreed or agreed strongly to it in the feasibility survey. All data analyses were done using SPSS version 25 (https://www.ibm.com/analytics/spss-statistics-software).

## Results

### Interrater Reliability

Table 2 shows the percentage exact agreement and interrater reliability for all items. The percentage exact agreement was on average 69% for the 6 Policies Subscale items and on average 83% for the 9 Prescriber Practices Subscale items. The ICC was excellent (0.91) for the Policies Subscale and on average good (0.84) for the 6 Policies Subscale items (range 0.75 to 0.93). For the 9 Prescriber Practices Subscale items, kappas were good (range 0.61 to 0.69) for 4, fair (range 0.46 to 0.57) for 3, and poor (range − 0.02 to 0.36) for 2. The percentage exact agreement and interrater reliability for the criteria for all items are reported in detail in Table 4 in the Online Appendix, which shows that the exact agreement was 80% or above for 37 (80%) of the 46 criteria. Kappa was excellent (range 0.75 to 1.00) for 9 criteria, good (range 0.61 to 0.72) for 12, fair (0.42 to 0.59) for 17 and poor (range − 0.05 to 0.38) for 8 criteria.

### Frequency Distribution

Table 3 shows descriptive statistics for each item, the Policies and Prescriber Practices subscales, and the total fidelity scale at 0 and 18 months. The table also shows distributions at 18 months regarding poor, adequate and full fidelity. Some items show a reasonably good variance after 18 months, but for items on shared decision-making policy, systematic monitoring of symptoms, systematic monitoring of side effects, and of somatic assessment at start of medication, fidelity ratings were consistently low. These are among the seven items without significant changes in fidelity over 18 months. The other eight items and the mean fidelity for the whole scale did show significant increases in fidelity.

**Table 3:** Descriptive statistics for items, subscales and fidelity scale (8 sites)

Fidelity scale items	0 months	18 months	Difference 0 and 18 months	Distribution of fidelity ratings at 18 months		
	Mean (SD)	Mean (SD)	Significance p (paired t-test)	Poor 1–3	Adequate 4	Full 5
Policies items						
(1) Shared decision-making policy	1.00 (0.00)	1.63 (0.92)	.095	8	0	0
(2) Access to medication list	2.88 (1.36)	4.38 (0.52)	.020	0	5	3
(3) Monitor and improve adherence	2.50 (0.53)	3.50 (0.53)	.001	0	4	4
(4) Monitor effect of medication	1.00 (0.00)	2.38 (1.69)	.054	5	2	1
(5) Monitor side effects of medication	2.63 (0.74)	3.50 (0.93)	.087	4	3	1
(6) Monitor clinical course after medication	1.63 (0.52)	3.00 (1.20)	.020	5	2	1
Policies Subscale fidelity	1.94 (0.31)	3.06 (0.51)	< .001	8	0	0
Prescriber Practices items						
(7) Medication decisions and patient preferences	2.50 (1.41)	4.13 (0.99)	.010	1	4	3
(8) List of medications and dose levels are updated	4.00 (1.85)	3.88 (1.46)	.763	3	1	4
(9) Polypharmacy only during change of drug	3.63 (1.51)	3.88 (1.36)	.351	2	3	3
(10) Choice of drug according to guidelines	3.50 (1.31)	4.38 (0.92)	.021	2	1	5
(11) Dosage of drug according to guidelines	3.00 (1.07)	3.88 (0.99)	.006	2	4	2
(12) Systematic monitoring of symptoms	1.00 (0.00)	1.13 (0.35)	.351	8	0	0
(13) Systematic monitoring of side effects	1.63 (0.74)	2.88 (1.25)	.049	6	1	1
(14) Medication adherence support	2.38 (1.06)	3.75 (1.39)	.014	3	2	3
(15) Somatic assessment at start of medication	1.13 (0.35)	1.63 (0.92)	.227	8	0	0
Prescriber Practices Subscale fidelity	2.53 (0.79)	3.28 (0.77)	.001	8	0	0
Total mean fidelity	2.29 (0.44)	3.19 (0.52)	< .001	8	0	0

Internal consistency (Cronbach’s alpha) of the total fidelity scale at each of the four fidelity assessments ranged from unacceptable to fair (range 0.43 to 0.75). Alpha was unacceptable (range 0.13 to 0.38) for the Policies Subscale and fair to good (range 0.69 to 0.86) for the Prescriber Practices Subscale.

### Sensitivity to Change

**Fig. 1:**
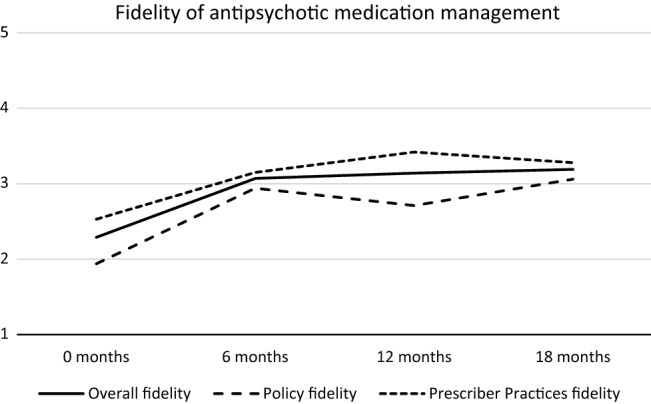
Development of fidelity overall and at policies and prescriber practices level

Figure 1 shows the development of fidelity across 18 months for the Policies and Prescriber Practices subscales and the total fidelity scale. The main change occurred from baseline to 6 months with little change from 6 to 18 months. At baseline, the mean site-level fidelity rating for the total scale was 2.29. By 6 months, mean fidelity had increased to 3.07, a significant increase of 0.78 (t =  − 6.62, p = 0.001). At 18 months, fidelity was 3.19, which was a nonsignificant increase of 0.12 (t =  − 0.98, p = 1.000) from 6 months. The increase of 0.90 in total fidelity from baseline to 18 months was significant (t =  − 9.11, p < 0.001) and with a large effect size (Cohen’s d_z_ = 2.66). The increase was also significant for both subscales, and Cohen’s d_z_ was 3.09 for the Policies Subscale and 1.79 for the Prescriber Practices Subscale. At no time period did the total mean fidelity exceed 4.00, the benchmark for adequate fidelity.

The Policies Subscale fidelity and the Prescriber Practices Subscale fidelity both showed a development parallel to the overall fidelity with the main increase during the first 6 months, but with the Policies Subscale fidelity below and the Prescriber Practices above the overall fidelity. The Pearson correlation between the Policies Subscale rating and the Prescriber Practices Subscale rating across all sites was moderate and negative (− 0.43 and − 0.45) at baseline and 6 months, and negligible (0.03 and 0.05) at 12 months and 18 months, respectively.

### Feasibility Survey

The 19 fidelity assessors reported that they spent on average 4.3 h (SD 1.3) on a fidelity visit, including an average of 2.9 h (SD 0.8) on reading and rating patient records. Altogether 89% agreed that the scale was clearly set out, 26% that it was easy to get information, 47% that it was easy to rate, 72% that it had good instructions, and 61% that it had good instructions for preparations. Regarding data sources, assessors reported interviews with clinicians and reading patient records were useful sources of information, while interviews with leaders were less useful.

## Discussion

The 15-item Antipsychotic Medication Management Scale operationalized evidence-based components from the research to assess the quality of antipsychotic medication management. The interrater reliability (ICC) was excellent for the Policies Subscale and good to excellent for the subscale items. The interrater reliability (kappa) for the Prescriber Practice Subscale items was on average fair and ranging from good to poor, but the percentage exact agreement was high, which may yield poor kappas because low frequency events result in a restriction of range in item ratings (Streiner et al. 2015). Sensitivity to change over time was excellent for the total scale and the two subscales. The distribution of site ratings at 18 months was good for half of the items, but none of the sites reached adequate fidelity level of 4.0 within 18 months. The overall picture was that the total scale and two subscales achieved adequate interrater reliability, though some item were not rated reliably.

The significant increase in total scale fidelity even with a small sample of eight sites showed that the scale is sensitive to change. Because the sites showed substantial improvement from baseline to 18 months, we conclude that this scale can reliably identify sites that improve adherence to clinical guidelines for evidence-based antipsychotic medication practices. Thus, this fidelity scale can be used to measure and guide implementation of the evidence-based model of antipsychotic medication management. The main increase during the first 6 months was during the 6 months with more intensive implementation support offered to clinicians and leaders through site visits every second week, after a joint one day workshop for clinicians and leaders from all experimental sites at the start.

To understand why the sites did not achieve adequate fidelity on several items and overall, despite training and implementation support, we considered the rating criteria and some comments from the fidelity assessors. Some evidence-based practice components, such as shared decision-making and systematic measurements of symptoms and side effects, represented a challenging culture shift in the mental health services in Norway. The culture in the services may change over time and implement these components, or the services may continue to reject these evidence-based components as unnecessary. Other components, such as considerations of phase of illness and somatic risks, may have been poorly documented. Some of the items may need better calibration, perhaps by making a few criteria less strict.

Internal consistency was mostly unacceptable for the total fidelity scale and the Policies Subscale, and fair to good for the Prescriber Practices Subscale. Because evidence-based practices often combine several discrete components, program performance on these different components may be uncorrelated, leading to poor internal consistency (Bond and Drake 2019). Thus, we may not expect to find a very high internal consistency among items in a fidelity scale. Still it is of interest to measure to what extent there is internal consistency for a group of components in an evidence-based practice, and in the current study the internal consistency was substantially higher for the Prescriber Practices Subscale than for the Polices Subscale.

While Taylor et al. (2009) found a moderate correlation between the prescriber and organizational fidelity in MedMAP, the current study found weak negative and negligible correlations between the Policies and Prescriber Practices subscales. This may indicate that medication management policy prescribed by the sites had limited impact on actual practice, which is one of the challenges in implementation and quality improvement (Shojania and Grimshaw 2005).

The fidelity assessors found the fidelity scale clearly set out and with good instructions, but it was difficult to find some of the information which contributed to some difficulty in rating items. This indicates that the fidelity scale is feasible, even if it was challenging to find some information. Good feasibility was also the experience of leaders at the eight sites, as they reported in individual telephone interviews at the end of the study that it was useful to get the fidelity assessments as feedback, that the assessments had been used actively to improve practice, and that the time used on the fidelity assessment was well spent.

Many previous studies have documented non-adherence to clinical guidelines, e.g., high rates of polypharmacy and low rates of using standardized scales, but few attempts to put guidelines for antipsychotic medication management together in a comprehensive scale exist. One exception, the MedMAP fidelity scale, also showed consistently low fidelity for use of rating scales assessing symptoms and side effects of antipsychotic medication (Howard et al. 2009; Taylor et al. 2009). The psychometric properties of our scale were mostly comparable to those of the MedMAP scale, but the average time for a fidelity visit was much shorter than for the MedMAP scale. As the average time for a fidelity visit in our study was less than a day, it would be possible to extend the fidelity visit for two additional hours so that both assessors could review all 10 patient records. Independent fidelity rating of all items would then make it possible to calculate ICC for all items, for both subscales and for the total fidelity scale.

### Limitations

Several limitations warrant mention. The Antipsychotic Medication Management Scale had minimal pilot testing. Some information was difficult to find in the patient records. Some ratings were not reliable. The numbers of sites were low.

## Conclusions and Implications

The Antipsychotic Medication Management Fidelity Scale is a new measure that shows good to fair interrater reliability, adequate sensitivity to change over time, and good feasibility. Use of the scale can assess fidelity to evidence-based guidelines for antipsychotic medication management and guide efforts to improve practice. Further research should improve and better calibrate some items, and improve the procedures for access to information.

## Electronic supplementary material

Below is the link to the electronic supplementary material.

Supplementary file1 (DOCX 38 kb)

Supplementary file2 (DOCX 24 kb)
