# Supplementary material for: The Antipsychotic Medication Management Fidelity Scale: Psychometric properties
Source: Adm Policy Ment Health. 2020 Feb 6;47(6):911–9. doi: 10.1007/s10488-020-01018-1 (PMC7547997; doi:10.1007/s10488-020-01018-1)
Supplement: Supplementary file 1 — Supplementary file1 (DOCX 38 kb) [file 10488_2020_1018_MOESM1_ESM.docx]

# The Antipsychotic Medication Management Fidelity Scale

Clinical unit (site): _____________________________­­­__________ Completed at ____­_________ (date ) by ______________________ (assessor)

**Brief instructions**

Procedure for fidelity assessment

- Fidelity assessment is done during one day by two fidelity assessors who are not working in the unit.
- The fidelity assessors have been trained in using the fidelity scale with the instructions and the summary sheet for the scale.
- The fidelity assessment is prepared and done as described in the instructions for preparation and implementation of the assessment.

Preparations for the fidelity assessment

- The unit must be clearly defined regarding which teams/services that are included in the site and the fidelity assessment.
- The unit manager prepares the program for the day in collaboration together with the assessor from the health trust.
- The unit manager identifies all relevant documents/procedures and send these to the assessor before the assessment.
- The unit manager makes arrangement with managers, clinicians and others for interviews during the assessment day.
- The unit manager ensures that print out of 10 randomly selected patient records are available as specified in the instructions for preparation and implementation of the assessment. These are drawn among patients with prescribed antipsychotic medication.

Tools for the fidelity assessment

- The fidelity scale with criteria (this document).
- Instructions and interview guide for fidelity assessment of physical health care at the system level (elements/items 1-6).
- Summary sheet for review and rating of patient records for fidelity assessment of physical health care at the individual level (elements/items 7-15).
- List of DDD (defined daily doses) and most important side effects of antipsychotic medications is included at the end of the summary sheet.

| **Items of Policies Subscale (use interview guide and form)** | | | | |
| --- | --- | --- | --- | --- |
| **1** | **Unit has a policy of shared decision making and has procedures to foster this** |  | Written material, observations and interview (interview guide) | 1. Unit gives all patients a brochure to all patients that explains what shared decision making is and how it is practiced in the unit. 2. Before medication consultations patients are given a brochure on shared decision making and a brief questionnaire to help them prepare their views. 3. Unit has procedures for documenting patient views and wishes in record notes from consultations on medication. 4. The unit uses a specific program to train physicians/clinicians in practicing shared decision making. 5. Participation in the training in shared decision making is required at least every second year, and participation is registered and documented.   **Scoring: Sum of criteria met (1 if 0-1 criteria met)** |
| **2** | **Unit has procedures to ensure that all stakeholders (physicians, other unit clinicians, GPs, and patients) have easy access to list of current patient medications** |  | Written material, observations and interview (interview guide) | 1. Unit has procedures to ensure patients receive a list of current medications with the current date when medication has been changed. 2. Unit has a defined location for storing patient medication list (e.g. specific place in paper records or electronic record). 3. Unit has a procedure for reviewing patient medication list at least every 6 months by the prescribing physician/clinician. 4. Medication lists are accessible to all clinicians in unit (including those on call). 5. Unit has a procedure for transmitting updated medication list to GP within seven days after medication change for outpatients, and within seven days after discharge of inpatients.   **Scoring: Sum of criteria met (1 if 0-1 criteria met)** |
| **3** | **Unit has procedures to monitor and improve patient adherence to prescribed medication** |  | Written material, observations and interview (interview guide) | 1. Unit gives all patients a brochure that explains the importance of adherence. 2. Unit uses a standardized patient self-report form or other method to assess medication adherence at each consultation on medication. 3. Physician/clinician shall in medication consultations ask how the patient takes the medication, and especially explore reasons if patient is not taking the medication as prescribed. 4. Unit support medication adherence (e. g. explore reasons for lack of adherence, supply dosage box, teach using app or other methods of reminders, and possibly offer use of depot injection).   **Scoring: Sum of criteria met + 1** |
| **4** | **Unit has procedures to monitor effectiveness of antipsychotic medications** |  | Written material, observations and interview (interview guide) | 1. The unit has decided to use of a specific symptom rating scale to assess the effect of antipsychotic medication. 2. The unit has a procedure that physicians/clinicians shall use the symptom rating scale for systematic assessment of symptoms at medication consultations. 3. At least 50 % of physicians/clinicians have within the last year completed initial or booster session of training in use of the symptom scale. 4. Physician/clinician gives feedback to the patient on the assessment of effect, preferably as a graph showing degree of change.   **Scoring: Sum of criteria met + 1** |
| **5** | **Unit has procedures to monitor side effects of medication and keep side effects lowest possible** |  | Written material, observations and interview (interview guide) | 1. The unit uses a specific checklist to identify serious side effects which may occur when using the prescribed medication. 2. The unit has a procedure that physicians /clinicians shall assess side effects of antipsychotic medication at each consultation on medication. 3. If significant side effects are identified, measures are taken to reduce these. 4. The unit has a procedure that the effect of measures to reduce side effects shall be documented in patient records within four weeks.   **Scoring: Sum of criteria met + 1** |
| **6** | **Unit has procedures to monitor patient clinical course after ending medication** |  | Written material, observations and interview (interview guide) | 1. The unit has decided to follow up the clinical course of patients for two years after antipsychotic medication has been ended. 2. The unit has a procedure to engage patients, carers/relatives and GP/other services in knowing early signs of relapse and actions to take if such signs. 3. The unit has a procedure to give reasons for and offer a follow-up for two years after ending antipsychotic medication, and with systematic tracking at least each six months to follow the clinical course of the patient. 4. If the patient prefer follow-up by the GP or other services, the unit has a procedure to offer support and collaboration to the GP/other service with reminders for systematic tracking at least each six months for two years.   **Scoring: Sum of criteria met + 1** |

| **Items of the Prescriber Practices Subscale (use summary sheet for review of patient records)** | | | | |
| --- | --- | --- | --- | --- |
| **7** | **Medication decisions incorporate patient own preferences and goals for medication** |  | Ten randomly selected patient records (see guidelines for extraction) | Documentation in patient records that patient preferences and goals for medication has been discussed at least once, and notes refer what the patient said about preferences and goals.  **Procedure: Documentation in a patient record is considered as passed if the criterion above is met.**  **Scoring: ≥ 8 charts pass = 5, 6-7 charts= 4, 4-5 charts = 3, 2-3 charts = 2, 0-1 charts=1.** |
| **8** | **List of medications and dose levels are updated at least every 6 months** |  | Ten randomly selected patient records (see guidelines for extraction) | The patient record includes a list of medications with the date when the list was last updated, and the list has been updated within the last 6 months.  **Procedure: Documentation in a patient record is considered as passed if the criterion above is met.**    **Scoring: ≥ 8 charts pass = 5, 6-7 charts= 4, 4-5 charts = 3, 2-3 charts = 2, 0-1 charts=1.** |
| **9** | **Polypharmacy avoided except during change of medication** |  | Ten randomly selected patient records (see guidelines for extraction) | The patient is prescribed only one antipsychotic drug, or two drugs with gradual tapering/increasing dosage during a specified time for changing from one drug to another, or two drugs with a clearly stated reason why polypharmacy is accepted.  **Procedure: Documentation in a patient record is considered as passed if the criterion above is met.**  **Scoring: ≥ 8 charts pass = 5, 6-7 charts= 4, 4-5 charts = 3, 2-3 charts = 2, 0-1 charts=1.** |
| **10** | **Choice of antipsychotic drug is in accordance with the guidelines for situation and phase of illness** |  | Ten randomly selected patient records (see guidelines for extraction) | The current (if continued) or new antipsychotic meets one of the following criteria:   - The antipsychotic drug has good effect and acceptable side effects and is continued. - Patient is prescribed an antipsychotic drug for the first time. - A new antipsychotic drug is prescribed after a first drug did not have effect and/or was not tolerated. - Clozapine is offered after two antipsychotic drugs have been tried with adequate dose for 8 weeks without having effect.   **Procedure: Documentation in a patient record is considered as passed if one of the criteria above is met.**  **Scoring: ≥ 8 charts pass = 5, 6-7 charts= 4, 4-5 charts = 3, 2-3 charts = 2, 0-1 charts=1.** |
| **11** | **Dose of antipsychotic drug is in accordance with the guidelines for situation and phase of illness** |  | Ten randomly selected patient records (see guidelines for extraction) | One of the following criteria is met regarding dose of antipsychotic drug:   - If first episode psychosis: Start dose in lowest dose range. Slow increase to maximum 2 DDD after 8 weeks. - Changing dose: A chosen dose is given for at least 6 weeks before the effect was evaluated and decision on medication and/or dose revised. - Acute phase at relapse: Dose is started as for first episode psychosis, but a higher maximum dose may be given. Take into account experiences from earlier psychosis. - Maintenance dose: Documented efforts to keep the dose as low as possible, and lower than the dose in the acute phase.   **Procedure: Documentation in a patient record is considered as passed if one of the criteria above is met.**  **Scoring: ≥ 8 charts pass = 5, 6-7 charts= 4, 4-5 charts = 3, 2-3 charts = 2, 0-1 charts=1.** |
| **12** | **Systematic monitoring of symptoms** |  | Ten randomly selected patient records (see guidelines for extraction) | Progress note from the most recent assessment of antipsychotic medication includes symptom ratings using the symptom rating scale which the unit has decided to use.  **Procedure: Documentation in a patient record is considered as passed if the criterion above is met.**  **Scoring: ≥ 8 charts pass = 5, 6-7 charts= 4, 4-5 charts = 3, 2-3 charts = 2, 0-1 charts=1.** |
| **13** | **Systematic monitoring of side effects** |  | Ten randomly selected patient records (see guidelines for extraction) | The patient record includes documentation for at least 3 of the 4 criteria below. See the list of side effects for the antipsychotic drugs (attached to the form).   - Side effects relevant for the antipsychotic drug have been assessed - Rating of side effect has been done using the rating scale decided by the unit - Side effects have been discussed with patient, or no side effects - Measures are taken to reduce any side effects, or no side effects   **Procedure: Documentation in a patient record is considered as passed if at least 3 of the 4 criteria above are met.**  **Scoring: ≥ 8 charts pass = 5, 6-7 charts= 4, 4-5 charts = 3, 2-3 charts = 2, 0-1 charts=1.** |
| **14** | **Support given for adherence to medication** |  | Ten randomly selected patient records (see guidelines for extraction) | The patient record includes documentation for at least 3 of the 4 criteria below.   - Adherence of prescribed medication is assessed together with the patient - Reasons for any lack of adherence is explored, or no lack of adherence - The importance of adherence is discussed, or no lack of adherence - Practical measures to improve adherences are done (offered depot injection if low adherence?), or no lack of adherence.   **Procedure: Documentation in a patient record is considered as passed if at least 3 of the 4 criteria above are met.**  **Scoring: ≥ 8 charts pass = 5, 6-7 charts= 4, 4-5 charts = 3, 2-3 charts = 2, 0-1 charts=1.** |
| **15** | **Somatic assessment before starting or changing medication** |  | Ten randomly selected patient records (see guidelines for extraction) | At start of any new antipsychotic medication or change of drug, the patient record documents a somatic examination and review of the illness history (or referrals in to earlier progress notes about this).   - Review of patient’s own history and family history of diabetes and heart disease - Registration of blood pressure, weight and BMI - Central metabolic measurements (blood lipids) - EKG done if this is recommended for the selected drug, or if history/examination indicates possible heart disease, or no documented indications for EKG.   **Procedure: Documentation in a patient record is considered as passed if at least 3 of the 4 criteria above are met.**  **Scoring: ≥ 8 charts pass = 5, 6-7 charts= 4, 4-5 charts = 3, 2-3 charts = 2, 0-1 charts=1.** |
