# Supplementary material for: The Antipsychotic Medication Management Fidelity Scale: Psychometric properties
Source: Adm Policy Ment Health. 2020 Feb 6;47(6):911–9. doi: 10.1007/s10488-020-01018-1 (PMC7547997; doi:10.1007/s10488-020-01018-1)
Supplement: Supplementary file 2 — Supplementary file2 (DOCX 24 kb) [file 10488_2020_1018_MOESM2_ESM.docx]

**Table 4 (online)*. Percentage exact agreement and interrater reliability** for items and criteria: Independent pairs of rating of 8 sites for items 1-6 and for 57 patient records for items 7-15**

| **#** | **Items (1-15) and criteria (a-e)** | | | **Agreement** | **ICC/Kappa** |
| --- | --- | --- | --- | --- | --- |
|  | **Policies Subscale (items 1-6)** | | |  |  |
| **1** | **Shared decision-making policy** | | | **66 %** | **0,75** |
| a | Give brochure to patients | | | 88 % | 0.59 |
| b | Give brochure and questionnaire before med consultations | | | 100 % | 1.00 |
| c | Document patient desires and goals in consultation record notes | | | 84 % | 0.57 |
| d | Shared decision-making training program | | | 84 % | 0.63 |
| e | Clinicians are trained in shared decision making every other year | | | 94 % | 0.00 |
| **2** | **Access to medication list** | | | **69 %** | **0.85** |
| a | Patients receive a list | | | 88 % | 0.75 |
| b | Location identified for med list storage | | | 94 % | 0.47 |
| c | Clinician reviews med list every 6 months | | | 72 % | 0.44 |
| d | All clinicians have access to list | | | 97 % | 0.78 |
| e | Transmit updated med list to GP within 7 days of discharge/change | | | 94 % | 0.72 |
| **3** | **Monitor and improve adherence** | | | **81 %** | **0.76** |
| a | Give brochure for adherence | | | 84 % | -0.05 |
| b | Assess adherence at consultations with a patient self-report | | | 84 % | 0.45 |
| c | Clinicians ask patients how they take their medications | | | 94 % | 0.76 |
| d | Unit supports medication adherence | | | 94 % | 0.48 |
| **4** | **Monitor effect of medication** | | | **72 %** | **0.93** |
| a | Unit uses a symptom rating scale | | | 91 % | 0.78 |
| b | Systematic procedure for using symptom rating scale | | | 88 % | 0.64 |
| c | 50% of clinicians received training on symptom scale last year | | | 94 % | 0.85 |
| d | Clinicians give assessment feedback | | | 88 % | 0.43 |
| **5** | **Monitor side effects of medication** | | | **56 %** | **0.87** |
| a | Clinician uses side effects checklist | | | 91 % | 0.71 |
| b | Assesses side effects at each consultation | | | 72 % | 0.42 |
| c | Reduces side effects if identified | | | 100 % | 1.00 |
| d | Documents side effect reduction in patient chart within 4 weeks | | | 81 % | 0.61 |
| **6** | **Monitor clinical course after medication** | | | **69 %** | **0.88** |
| a | Unit follows up on clinical course for 2 years after medication end | | | 97 % | 0.89 |
| b | Advocacy procedure for relapse identification and action plans | | | 78 % | 0.45 |
| c | Unit gives reasons for ending medication, and follow-up | | | 88 % | 0.45 |
| d | Collaborates with external services for follow-ups as patient prefers | | | 88 % | 0.67 |
|  | **Prescriber Practices Subscale (items 7-15)** | | |  |  |
| **7** | **Medication decisions and patient preferences** | | | **81 %** | **0.61** |
| **8** | **List of medications and dose levels are updated** | | | **89 %** | **0.67** |
| **9** | **Polypharmacy only during change of drug** | | | **86 %** | **0.69** |
| **10** | **Choice of drug according to guidelines** | | | **70 %** | **0.36** |
| a | Drug has good effect and acceptable side effects | | | 74 % | 0.47 |
| b | Clinician prescribed first antipsychotic drug | | | 100 % | 0.00 |
| c | Prescribed second drug after first with no effect or not tolerated | | | 91 % | 0.71 |
| d | Prescribed Clozapine after two drugs with no effect after 8 weeks | | | 100 % | 0.00 |
| **11** | **Dosage of drug according to guidelines** | | | **79 %** | **0.51** |
| a | Start dose in low range, slowly increase to max 2 DDD in 8 weeks | | | 97 % | 0.65 |
| b | A dose is given at least 6 weeks before reevaluation and change | | | 90 % | 0.51 |
| c | For acute phase at relapse, a dose is started with higher maximum | | | 93 % | 0.30 |
| d | Clinician documents efforts to keep dosage as low as possible | | | 65 % | 0.30 |
| **12** | **Systematic monitoring of symptoms** | | | **95 %** | **-0.02** |
| **13** | **Systematic monitoring of side effects** | | | **77 %** | **0.46** |
| a | Relevant side effects assessed | | | 75 % | 0.50 |
| b | Unit uses decided rating scale to rate side effects | | | 95 % | 0.38 |
| c | Clinician discusses side effects with patient, or no side effects | | | 86 % | 0.72 |
| d | Measures to reduce side effects taken, or no side effects | | | 77 % | 0.54 |
| **14** | **Medication adherence support** | | | **81 %** | **0.61** |
| a | Clinician assesses adherence with patient | | | 81 % | 0.61 |
| b | Explores lack of adherence, or no lack of adherence | | | 75 % | 0.51 |
| c | Discuss importance of adherence, or no lack of adherence | | | 79 % | 0.58 |
| d | Measures to improve adherence taken, or no lack of adherence | | | 83 % | 0.64 |
| **15** | **Somatic assessment at start of medication** | | | **91 %** | **0.60** |
| a | Clinician reviews history of diabetes and heart disease | | | 90 % | 0.44 |
| b | Register blood pressure, weight, and BMI at start of medication | | | 81 % | 0.36 |
| c | Metabolic measurements (blood lipids) at start of medication | | | 86 % | 0.65 |
| d | EKG if recommended, or no EKG recommendations | | | 95 % | 0.86 |
|  | **Summary for items (like in Table 2):** | | |  |  |
|  | **Average agreement and ICC for items 1-6 (Policies)** | | | **69 %** | **.84** |
|  | **Average agreement and kappa for items 7-15 (Practices)** | | | **83 %** | **.50** |
|  | **Summary for criteria:** | | |  |  |
|  | **Average for criteria of Policies Subscale** | | | **89 %** | **.60** |
|  | **Average for criteria of Prescriber Practices Subscale** | | | **86 %** | **.49** |
|  | **Average for criteria of the whole fidelity scale** | | | **87 %** | **.55** |
|  | | | | | |
|  | **Distribution of ratings of criteria** | | | | |
|  | **Exact agreement** | **Policies criteria** | **Prescriber Practices** | **Total fidelity scale** | |
|  | 60- 69 % | 0 | 1 | 1 | |
|  | 70-79 % | 3 | 5 | 8 | |
|  | 80-89 % | 11 | 5 | 16 | |
|  | 90-100 % | 12 | 9 | 21 | |
|  | **Cohen’s Kappa** | **Policies criteria** | **Prescriber Practices** | **Total fidelity scale** | |
|  | Poor (< .21) | 2 | 2 | 4 | |
|  | Fair (.21 - .40) | 0 | 4 | 4 | |
|  | Moderate (.41 - .60) | 10 | 7 | 17 | |
|  | Substantial (.61 - .74) | 6 | 6 | 12 | |
|  | Excellent (.75 – 1.00) | 8 | 1 | 9 | |

*) Table 4 also contains the results for items reported in Table 2 in the paper, so that these here in Table 4 may be seen together with the results for the criteria.

**) ICC for items rated 1-5 and Cohen’s Kappa for patient records rated passed/failed and for criteria rated passed/failed.
